# Employment Situation of Parents of Long-Term Childhood Cancer Survivors

**DOI:** 10.1371/journal.pone.0151966

**Published:** 2016-03-18

**Authors:** Luzius Mader, Corina S. Rueegg, Janine Vetsch, Johannes Rischewski, Marc Ansari, Claudia E. Kuehni, Gisela Michel, Swiss Paediatric Oncology Group (SPOG)

**Affiliations:** 1 Department of Health Sciences and Health Policy, University of Lucerne, Lucerne, Switzerland; 2 Department of Oncology/Hematology, Children’s Hospital, Cantonal Hospital Lucerne, Lucerne, Switzerland; 3 Unit of Oncology/Hematology, Department of Pediatric, Geneva University Hospital, Geneva, Switzerland; 4 Institute of Social and Preventive Medicine, University of Bern, Bern, Switzerland; Aichi Cancer Center Research Institute, JAPAN

## Abstract

**Background:**

Taking care of children diagnosed with cancer affects parents’ professional life. The impact in the long-term however, is not clear. We aimed to compare the employment situation of parents of long-term childhood cancer survivors with control parents of the general population, and to identify clinical and socio-demographic factors associated with parental employment.

**Methods:**

As part of the Swiss Childhood Cancer Survivor Study, we sent a questionnaire to parents of survivors aged 5–15 years, who survived ≥5 years after diagnosis. Information on control parents of the general population came from the Swiss Health Survey (restricted to men and women with ≥1 child aged 5–15 years). Employment was categorized as not employed, part-time, and full-time employed. We used generalized ordered logistic regression to determine associations with clinical and socio-demographic factors. Clinical data was available from the Swiss Childhood Cancer Registry.

**Results:**

We included 394 parent-couples of survivors and 3’341 control parents (1’731 mothers; 1’610 fathers). Mothers of survivors were more often not employed (29% versus 22%; p_trend_ = 0.007). However, no differences between mothers were found in multivariable analysis. Fathers of survivors were more often employed full-time (93% versus 87%; p_trend_ = 0.002), which remained significant in multivariable analysis. Among parents of survivors, mothers with tertiary education (OR = 2.40, CI:1.14–5.07) were more likely to be employed. Having a migration background (OR = 3.63, CI: 1.71–7.71) increased the likelihood of being full-time employed in mothers of survivors. Less likely to be employed were mothers of survivors diagnosed with lymphoma (OR = 0.31, CI:0.13–0.73) and >2 children (OR = 0.48, CI:0.30–0.75); and fathers of survivors who had had a relapse (OR = 0.13, CI:0.04–0.36).

**Conclusion:**

Employment situation of parents of long-term survivors reflected the more traditional parenting roles. Specific support for parents with low education, additional children, and whose child had a more severe cancer disease could improve their long-term employment situation.

## Introduction

The intensive and long lasting experience of a cancer diagnosis during childhood and its treatment places the whole family at risk for compromised functioning with long-term social and financial consequences [1–4]. Parents face difficulties in managing everyday domestic responsibilities, work life, and care taking for the diseased child and other family members, often for considerable periods of time [1, 3–5]. As a consequence, parents’ employment situation may be affected during the years of and after treatment. One or both of the parents may take time off work, reduce or leave paid employment completely to care for the sick child during treatment [1, 3, 5–10]. Changes in employment after the diagnosis have been shown to be more pronounced among mothers [1, 3, 8, 11], because they were more often in charge of staying with the child during treatment and medical care [1].

Previous studies suggested that disruptions of parents’ employment situation are most frequent shortly after diagnosis and during treatment [1, 3, 5, 7–11]. However, extended absences during the child’s treatment may affect the parents ability to return to their previous employment level [1]. Active cancer treatment involving frequent hospitalizations for several years is followed by regular follow-up appointments, which may be time consuming for parents and interfere with their employment situation over a long time. Generally, interruptions in employment may have considerable long-term consequences on professional life such as lack of skill development, lost networking, or missed opportunities for promotion [12]. In this study, we aimed to 1) describe the employment situation of parents of long-term childhood cancer survivors compared to control parents of the Swiss general population, and 2) identify socio-demographic and cancer-related factors (parents of survivors only) associated with parental employment.

## Materials and Methods

### Ethics statement

Ethical approval was provided through the general cancer registry permission of the Swiss Childhood Cancer Registry (SCCR; Swiss Federal Expert Commission for Professional secrecy in Medical Research) and a non-obstat statement was obtained from the ethics committee of the canton of Bern, stating that no additional informed consent was necessary for the Swiss Childhood Cancer Survivor Study (SCCSS). Information regarding parents of survivors from the SCCSS and control parents from the Swiss Health Survey (SHS) was anonymized prior to data analysis.

### The Swiss Childhood Cancer Survivor Study (SCCSS)

The SCCR registers all children and adolescents younger than 21 years who were diagnosed with leukemia, lymphoma, central nervous system (CNS) tumor, malignant solid tumors or Langerhans cell histiocytosis (LCH) in Switzerland [13, 14]. The SCCSS is a nationwide, long-term follow-up study of all patients registered in the SCCR, diagnosed between 1976–2005, aged <21 years at diagnosis, who survived ≥5 years [15]. All childhood cancer survivors eligible for the SCCSS were contacted with a questionnaire between 2007 and 2012. For survivors aged ≤15 years parents were asked to complete the questionnaire for their child between 2010 and 2011 [15]. For the current study, we only included information from the questionnaires to parents of survivors aged ≤15 years. For each survivor, the parent-couple completed one questionnaire including questions addressed to the mother and father. Non-responders received a reminder letter with a second copy of the questionnaire. If no response was obtained after the reminder letter, non-responders were contacted by phone. The questionnaire was available in German, French, and Italian.

### Comparison group

We used data of the SHS 2012 as comparison group. The SHS is a computer-assisted telephone survey of a random sample of Swiss residents aged ≥15 years conducted every 5 years. It provides data on the health state, health behavior, use of health services and socio-demographic information of the Swiss population [16, 17]. Details about the data collection in the SHS are described elsewhere [17]. Briefly, a random weighted sample was obtained by stratified selection on two stages: First, households were randomly selected from each canton, and second, one person was randomly selected in each household for the telephone interview. In total, 21’597 interviews were realized (response rate 53%) [17]. The SHS sample consisted of independent adult persons who answered only questions for themselves, not for their partner.

### Measurements

The questionnaire of the SCCSS was created based on the questionnaires used in the childhood cancer survivor studies of the US and UK [18, 19]. In addition, questions on socio-economic measures were added from the Swiss Health Survey 2007 [20] and the Swiss Census [21].

### Outcome measure

#### Employment situation

Parents of survivors and control parents were asked about their current employment situation using similar questions with the response options (more than one answer possible): full-time, part-time, more than one part-time job, not employed, homemaker, in education, and invalidity pension. Employment situation was categorized as “not employed”, “part-time”, and “full-time”. Having more than one part-time job was categorized as “part-time”. Participants who indicated homemaker, in education, or invalidity pension without being employed full- or part-time were categorized as “not employed”.

### Explanatory variables

#### Socio-demographic variables

The following socio-demographic variables were assessed for parents of survivors and control parents: age at study, migration background, language region, education, and number of children. Parents’ age at study was categorized into “<40 years”, “40–45 years”, “45–50 years”, and “>50 years”. Parents were defined as having a migration background if they were not Swiss citizen since birth or moved to Switzerland after birth. Language region was categorized into “German” and “French/Italian”. Education was divided into three categories: “primary” (compulsory schooling including vocational training/apprenticeship); “secondary” (high school, teacher schools, technical and commercial schools, etc.); “tertiary” (university degree) [22]. The number of children was divided into “≤2 children” and “>2 children”. The age at study of the child with cancer was categorized into “<9 years”, “9–12 years”, and “>12 years”.

#### Cancer-related variables

For parents of survivors, we extracted clinical data of their child from the SCCR including: age at diagnosis, diagnosis, treatment, time since diagnosis, and relapse status. Age at diagnosis was divided into: “<2 years”, “2–5 years”, and “>5 years”. Diagnosis was coded according to the International Classification of Childhood Cancer—Third Edition (ICCC-3) [23]. For the analysis, diagnosis was categorized into: “leukemia”, “lymphoma”, “CNS tumors”, “soft tissue sarcoma/bone tumor”, and “other tumors”. Treatment modalities were coded hierarchically into: “surgery only”, “chemotherapy” (may have had surgery), “radiotherapy” (may have had surgery and/or chemotherapy), and “stem cell transplantation” (SCT). Time since diagnosis was divided into three categories: “<8 years”, “8–11 years”, and “>11 years”. Relapse status was coded as “yes” or “no”. Parents self-reported in the questionnaire if their child suffered from late effects (defined as physical or psychological problems caused by the cancer and/or its treatment) categorized as “yes” or “no”.

#### Statistical analysis

All analyses were performed using Stata, version 13.1 (StataCorp LP, College Station, TX). We included only survivors living with both parents or with one parent and a new partner. The sample of the SHS population was restricted to participants living in a couple household with at least one child aged 5–15 years. Single parents and parents >65 years (age of retirement in Switzerland) were excluded in both populations. Mothers and fathers in the SHS population were younger, more often migrants, from French- or Italian language region, of tertiary education and had fewer children (S1 Table). To account for these differences we standardized mothers and fathers of the SHS population on age (categorical), migration background, language, education, and number of children according to the marginal distribution in mothers and fathers of survivors, respectively. To calculate appropriate weights for this standardization, we used multivariable logistic regression with being a control parent as outcome separately for mothers and fathers [24, 25]. The weight for parents of survivors was set to one. All analyses were based on the weighted SHS population using the *survey* command in Stata. Stata’s *survey* command fits statistical models for complex survey data by adjusting the results of a command for previously defined survey settings such as the weights for the SHS population [26].

First, we used a combined dataset of parents of survivors and control parents to compare the distribution of employment situation using chi-squared tests and tests for trend across different categories of employment. For parents of survivors, we additionally stratified employment by main diagnostic group. We then fitted univariable and multivariable generalized ordered logistic regression models for ordinal dependent variables to determine associations between employment situation and socio-demographic variables in the combined dataset. Ordered logistic regression was used if the proportional odds assumption was met for the respective model. We used the *autofit* option of Stata’s *gologit2* command to identify the partial proportional odds model that best fitted the data. We provided separate estimates for the different categories of employment (for those not employed in contrast to those part- or full-time employed; for those not employed or part-time employed in contrast to those full-time employed) if the proportional odds assumption was not met in the respective model. We used interaction tests to determine whether associations differed between parents of survivors and control parents. Second, we investigated associations with socio-demographic and cancer-related variables in univariable and multivariable generalized ordered logistic regression models in parents of survivors only. All variables significantly (p<0.05) associated with the employment situation of mothers or fathers in the univariable regression were included in multivariable analyses. “Not employed” was used as the reference group in all regression analyses. We used Wald tests to calculate global p-values. In a sensitivity analysis we used a multilevel modelling approach to control for family clustering as it may be expected that decisions regarding the employment situation of mothers and fathers are made at a family rather than an individual level. However, there was no indication that family clustering was relevant in our analyses such that we show separate analyses for mothers and fathers.

## Results

### Characteristics of the study population

**Fig 1:**
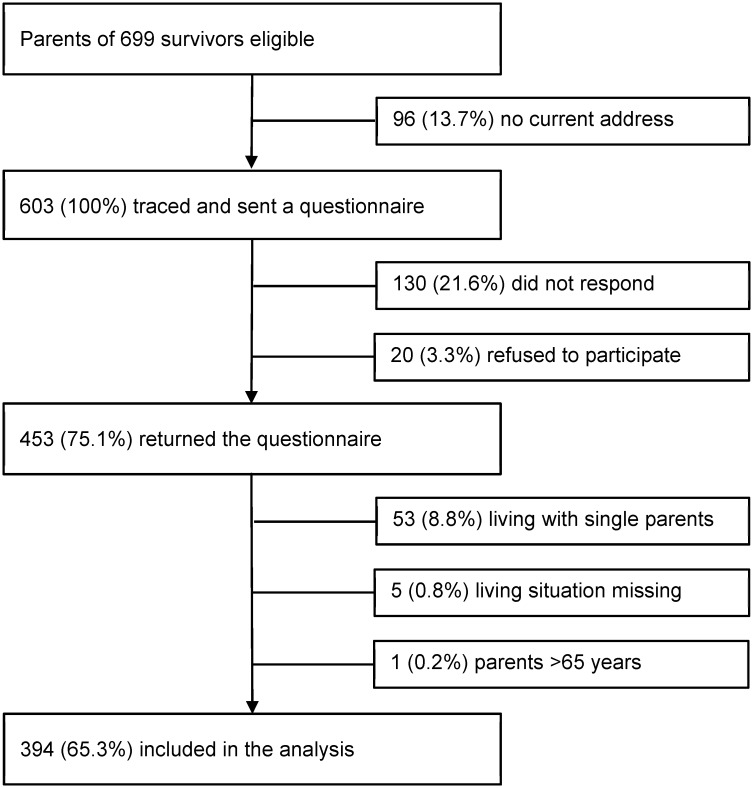
Participants of the parents questionnaire of the Swiss Childhood Cancer Survivor Study. Flow diagram of our study population starting from those eligible in the Swiss Childhood Cancer Registry to those included in the analysis.

Of 699 eligible survivors, 603 parents could be contacted (Fig 1). Of those, 453 (75.1%) returned the questionnaire. Parents of survivors were excluded if the survivor was living with a single parent (N = 53, 8.8%), if information on living situation was missing (N = 5, 0.8%), or if parents were >65 years (N = 1, 0.2%), resulting in a final sample of 394 mothers and fathers of survivors. In the SHS population (N = 21’597, who completed the interview), we excluded mothers and fathers not living in a couple household (N = 12’739, 31.3%), with no child aged 5–15 years (N = 5’503, 13.5%), and who were >65 years (N = 14, 0.03%). The final SHS population consisted of 3’341 eligible participants: 1’731 mothers; 1’610 fathers (S1 Fig).

The mean age of participating mothers and fathers of survivors was 42.7 years (Standard deviation (SD) = 4.8) and 45.8 years (SD = 5.8), respectively (Table 1). A migration background was reported by 30.0% of mothers and 24.1% of fathers. Sixty-nine percent were from a German-speaking region of Switzerland. The mean number of children was 2.5 (SD = 1.0). The SHS population was standardized for the above mentioned socio-demographic variables. The mean age of survivors at study was 12.0 years (SD = 2.7). Most children were diagnosed with leukemia (36.6%), followed by CNS tumors (16.8%), and neuroblastoma (8.6%). Mean time since diagnosis was 9.0 years (SD = 2.5). A cancer relapse was observed in 11.2%, and 39.4% of parents reported that their child suffered from late effects.

**Table 1:** Characteristics of parents of survivors and weighted control parents.

	Parents of survivors (N = 394)				Control parents^a^ (N = 3’341)						
	Mothers		Fathers		Mothers (N = 1’731)				Fathers (N = 1’610)		
	n	%^b^	n	%^b^	n		%^b^		n	%^b^	
**Characteristics of parents**											
*Age at study*											
<40 years	110	29.0	55	14.6		498	28.9	224		14.0	
40–45 years	140	36.9	130	34.4		629	36.4	550		34.3	
45–50 years	114	30.1	109	28.8		526	30.5	470		29.4	
>50 years	15	4.0	84	22.2		74	4.3	358		22.3	
*Migration background*											
No	276	70.1	299	75.9		1’261	73.1	1’238		77.3	
Yes	118	30.0	95	24.1		465	26.9	363		22.7	
*Language region*											
German	271	69.0	271	69.0		1’170	67.8	1’090		68.1	
French/Italian	122	31.0	122	31.0		556	32.2	511		31.9	
*Education*											
Primary	242	64.2	178	48.6		1’126	65.3	763		47.7	
Secondary	95	25.2	132	36.1		428	24.8	592		37.0	
Tertiary	40	10.6	56	15.3		172	10.0	246		15.4	
*Number of children*											
≤2 children	226	57.4	226	57.4		1’004	58.2	923		57.7	
>2 children	168	42.6	168	42.6		722	41.8	678		42.3	
**Characteristics of the survivor**	n		%^b^			n		%^b^			
*Age of child at study*											
<9 years	72		18.3			-		-			
9–12 years	104		26.4			-		-			
>12 years	218		55.3			-		-			
*Age of child at diagnosis*											
<2 years	167		42.4			-		-			
2–5 years	137		34.8			-		-			
>5 years	90		22.8			-		-			
*Diagnosis (ICCC-3)*											
Leukemia	144		36.6			-		-			
Lymphoma	29		7.4			-		-			
CNS tumor	66		16.8			-		-			
Neuroblastoma	34		8.6			-		-			
Retinoblastoma	31		7.9			-		-			
Renal tumor	33		8.4			-		-			
Hepatic tumor	8		2.0			-		-			
Bone tumor	6		1.5			-		-			
Soft tissue sarcoma	23		5.8			-		-			
Germ cell tumor	7		1.8			-		-			
Langerhans cell histiocytosis	11		2.8			-		-			
Other tumors^c^	2		0.5			-		-			
*Treatment*											
Surgery	65		16.6			-		-			
Chemotherapy	245		62.5			-		-			
Radiotherapy	62		15.8			-		-			
Stem cell transplantation	20		5.1			-		-			
*Time since diagnosis*											
<8 years	167		42.4			-		-			
8–11 years	141		35.8			-		-			
>11 years	86		21.8			-		-			
*Relapse*											
No	350		88.8			-		-			
Yes	44		11.2			-		-			
*Parent-reported late effects*											
No	217		60.6			-		-			
Yes	141		39.4			-		-			
	Mean	SD	Mean	SD		Mean	SD	Mean			SD
Age of parents at study	42.7	4.8	45.8	5.8		42.0	5.5	45.5			7.0
Age of child at study	12.0	2.7	12.0	2.7		-	-	-			-
Age of child at diagnosis	3.1	2.3	3.1	2.3		-	-	-			-
Time since diagnosis	9.0	2.5	9.0	2.5		-	-	-			-

Abbreviations: CNS, central nervous system; ICCC-3, International Classification of Childhood Cancer—Third Edition; n, number; SD, standard deviation.

^a^Calculated on weighted analysis (weights on age at study, migration background, language region, education, number of children separately for mothers and fathers).

^b^Percentages are based upon available data for each variable.

^c^Other malignant epithelial neoplasms, malignant melanomas, and other or unspecified malignant neoplasms.

### Employment situation of parents of survivors and control parents

**Fig 2:**
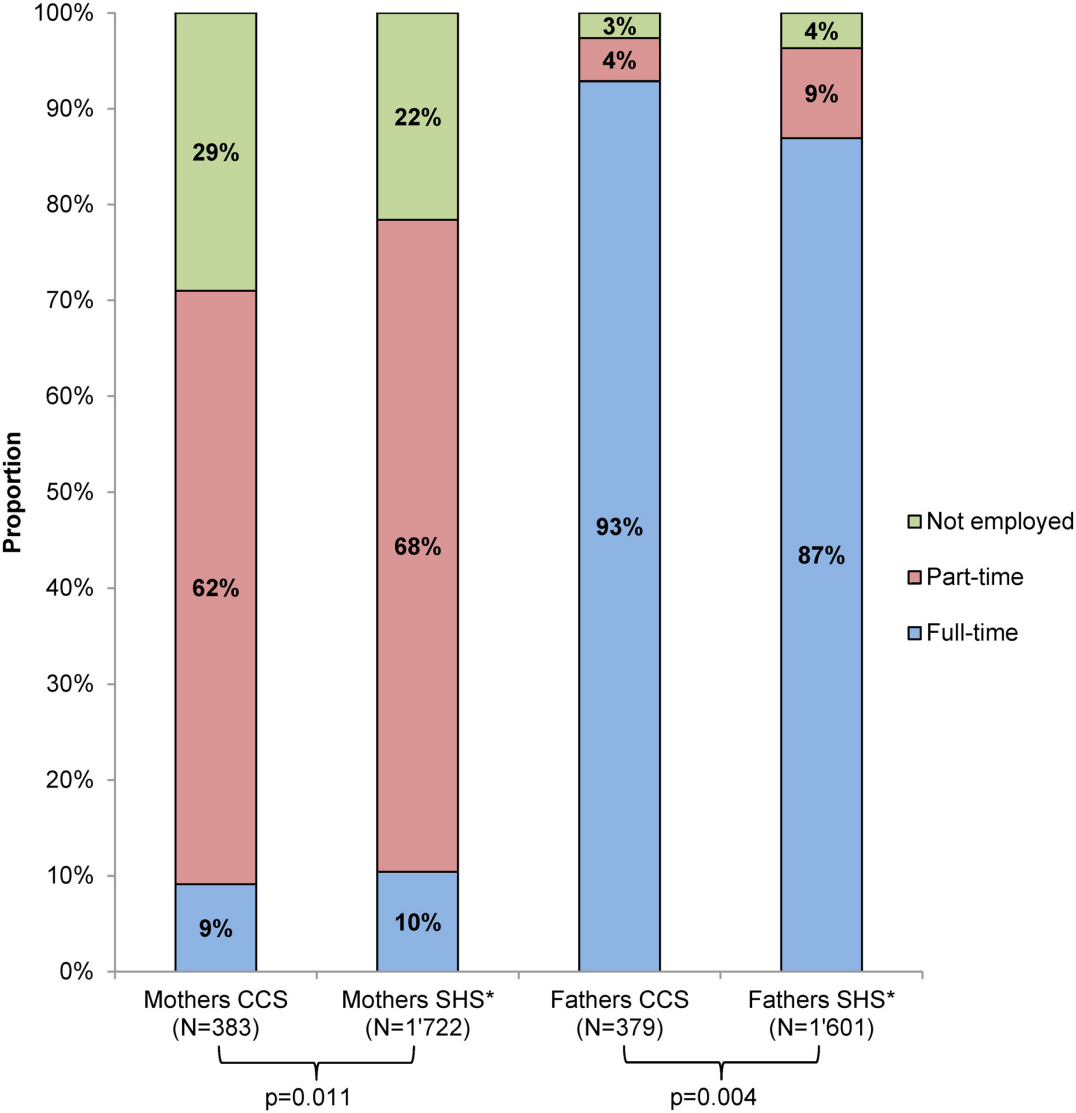
Employment situation of parents of survivors compared to control parents. Comparison of employment situation of mothers and fathers of survivors (CCS) and control mothers and fathers (SHS). The numbers in the figure represent the proportion of mothers and fathers who reported the respective employment situation. P-values were calculated from chi-square statistics comparing mothers of survivors with control mothers and fathers of survivors with control fathers. *Weighted proportions and numbers for control mothers and fathers according to age at study, migration background, language region, education, and number of children of mothers and fathers of survivors. Abbreviations: CCS, childhood cancer survivors; SHS, Swiss Health Survey.

Overall, the employment situation of parents of survivors was significantly different from control parents (Fig 2; p_mothers_ = 0.011; p_fathers_ = 0.004). In both populations the majority of mothers was employed part-time (mothers of survivors: 62%, control mothers: 68%). However, overall, more mothers of survivors were not employed compared to control mothers (29% vs. 22%; p_trend_ = 0.007). This pattern was observed across all diagnostic groups and was most pronounced for mothers of survivors diagnosed with lymphoma (S2 Fig); 48% of mothers of lymphoma survivors were not employed.

For fathers, we observed a different employment pattern. In both populations, most fathers were employed full-time (Fig 2; fathers of survivors: 93%, control fathers: 87%). However, overall, more fathers of survivors were employed full-time compared to control fathers (p_trend_ = 0.002). This pattern was observed across all diagnostic groups (S2 Fig). All fathers of survivors diagnosed with lymphoma were employed full-time.

### Associations with socio-demographic variables in parents of survivors and control parents

In univariable regression of the combined dataset including parents of survivors and control parents we found all socio-demographic variables to be associated with either the mothers’ or fathers’ employment situation (Table 2). We observed a significant interaction for the number of children and being a mother of a survivor or a control mother, such that more mothers of survivors with >2 children were not employed (OR = 0.46, CI:0.30–0.69) than control mothers of >2 children (OR = 0.76, CI:0.62–0.94, p_interaction_ = 0.022). In multivariable analysis among mothers, the associations with having >2 children (OR = 0.76, CI:0.60–0.95; including the interaction with being a mother of a survivor: p_interaction_ = 0.047); Table 3) remained statistically significant. In univariable analysis among mothers, we found associations with migration background, language region, and education that remained statistically significant in multivariable analysis. Mothers with a migration background were more often not employed (OR = 0.52, CI:0.40–0.67) or full-time employed (OR = 2.23, CI:1.55–3.23). Mothers from French/Italian language region were more likely to be full-time employed (OR = 2.01, CI:1.35–3.00). Mothers with secondary (OR = 1.39, CI:1.05–1.84) or tertiary education (OR = 2.21, CI:1.49–3.28) were more likely to be employed. The interaction for migration background was no longer significant in multivariable analysis (p = 0.158).

**Table 2:** Factors associated with employment from univariable generalized ordered^a^ logistic regression models^b^ (combined dataset including parents of survivors and control parents).

	Mothers (N = 2’125)					Fathers (N = 2’004)				
	n (%)	OR	95% CI	p-value^c^	p-value interaction^c^^,^^d^	n (%)	OR	95% CI	p-value^c^	p-value interaction^c^^,^^d^
*Population*				**0.007**					**0.002**	
Control parents	1'731 (81.5)	1.00				1’610 (80.3)	1.00			
Parents of survivors	394 (18.5)	0.73	0.57–0.92			394 (19.7)	1.94	1.27–2.97		
*Age at study*				0.262	0.912^e^				**0.002**	0.798
<40 years	609 (29.0)	1.00				282 (14.3)	1.00			
40–45 years	772 (36.7)	1.34	0.99–1.81			680 (34.4)	1.07	0.61–1.88		
45–50 years	637 (30.3)	1.23	0.90–1.69			576 (29.1)	1.04	0.59–1.84		
>50 years	87 (4.1)	1.04	0.63–1.71			441 (22.3)	0.48	0.27–0.85		
*Migration background*^f^				-	**0.042**				-	0.983
No	1’516 (71.5)	1.00				1’528 (76.6)	1.00			
Yes	604 (28.5)	0.72^g^	0.54–0.96	**0.025**		467 (23.4)	0.27^g^	0.15–0.52	**<0.001**	
		3.06^h^	2.10–4.47	**<0.001**			0.66^h^	0.45–0.97	**0.033**	
*Language region*^f^				-	0.203				0.401	0.684
German	1’450 (68.4)	1.00				1’367 (68.5)	1.00			
French/Italian	669 (31.6)	1.09^g^	0.82–1.45	0.562		627 (31.5)	0.85	0.58–1.24		
		2.28^h^	1.56–3.32	**<0.001**						
*Education*				**<0.001**	0.331				**0.020**	0.985^i^
										0.073^j^
Primary	1’361 (64.7)	1.00				947 (48.2)	1.00			
Secondary	526 (25.0)	1.35	1.03–1.77			719 (36.5)	1.27	0.85–1.89		
Tertiary	216 (10.3)	2.33	1.56–3.50			302 (15.3)	0.63	0.40–1.02		
*Number of children*				**<0.001**	**0.022**				0.447	0.323
≤2 children	1’224 (57.7)	1.00				1’147 (57.5)	1.00			
>2 children	896 (42.3)	0.58	0.46–0.74			848 (42.5)	1.15	0.80–1.65		

CI, confidence interval; n, number; OR, odds ratio. bold, p-value lower than 0.05.

^a^Ordering: not employed, part-time, full-time. OR<1 indicates that employment situation "not employed" is more likely. OR>1 indicates that employment situation "full-time/part-time" is more likely.

^b^Calculated on weighted analysis (weights on age at study, migration background, language region, education, number of children separately for mothers and fathers).

^c^Global p-value calculated from Wald tests.

^d^P-value for interaction between parents of survivors and control parents.

^e^Interaction for age group >50 years not calculated (numbers to low in mothers of survivors).

^f^Assumption of proportional odds not met for migration background (mothers and fathers) and language region (mothers only).

^g^OR for those not employed in contrast to those part- or full-time employed. OR<1 indicate that higher values on the explanatory variable increase the likelihood of being not employed. OR>1 indicates that higher values on the explanatory variable increase the likelihood of being part- or full-time employed.

^h^OR for those not employed or part-time employed in contrast to those full-time employed. OR<1 indicate that higher values on the explanatory variable increase the likelihood of being not employed or part-time employed. OR>1 indicates that higher values on the explanatory variable increase the likelihood of being full-time employed.

^i^Assumption of proportional odds not met for interaction. P-value for interaction for those not employed in contrast to those part- or full-time employed.

^j^Assumption of proportional odds not met for interaction. P-value for interaction for those not employed or part-time employed in contrast to those full-time employed.

**Table 3:** Factors associated with employment from multivariable generalized ordered^a^ logistic regression models^b^ (combined dataset including parents of survivors and control parents).

	Mothers (N = 2’125)					Fathers (N = 2’004)				
	n (%)	OR	95% CI	p-value^c^	p-value interaction^c^^,^^d^	n (%)	OR	95% CI	p-value^c^	p-value interaction^c^^,^^d^
*Population*				0.237	-				**0.042**	-
Control parents	1'731 (81.5)	1.00				1’610 (80.3)	1.00			
Parents of survivors	394 (18.5)	0.80	0.55–1.16			394 (19.7)	1.93	1.02–3.62		
*Age at study*				0.157	-				**0.002**	-
<40 years	609 (29.0)	1.00				282 (14.3)	1.00			
40–45 years	772 (36.7)	1.33	0.98–1.80			680 (34.4)	1.12	0.62–2.00		
45–50 years	637 (30.3)	1.19	0.86–1.64			576 (29.1)	1.08	0.61–1.93		
>50 years	87 (4.1)	0.82	0.48–1.42			441 (22.3)	0.49	0.27–0.88		
*Migration background*^e^				-	0.158					0.475
No	1’516 (71.5)	1.00				1’528 (76.6)	1.00			
Yes	604 (28.5)	0.52^f^	0.40–0.67	**<0.001**		467 (23.4)	0.23^f^	0.13–0.41	**<0.001**	
		2.23^g^	1.55–3.23	**<0.001**			0.67^g^	0.48–0.93	**0.017**	
*Language region*^e^				-	-				0.945	-
German	1’450 (68.4)	1.00				1’367 (68.5)	1.00			
French/Italian	669 (31.6)	1.02^f^	0.76–1.38	0.882		627 (31.5)	0.99	0.67–1.46		
		2.01^g^	1.35–3.00	**0.001**						
*Education*				**<0.001**	-				0.099	-
Primary	1’361 (64.7)	1.00				947 (48.2)	1.00			
Secondary	526 (25.0)	1.39	1.05–1.84			719 (36.5)	1.09	0.72–1.63		
Tertiary	216 (10.3)	2.21	1.49–3.28			302 (15.3)	0.65	0.40–1.05		
*Number of children*				**0.014**	**0.047**				0.725	0.196
≤2 children	1’224 (57.7)	1.00				1’147 (57.5)	1.00			
>2 children	896 (42.3)	0.76	0.60–0.95			848 (42.5)	1.06	0.76–1.49		

CI, confidence interval; n, number; OR, odds ratio. bold, p-value lower than 0.05.

^a^Ordering: not employed, part-time, full-time. OR<1 indicates that employment situation "not employed" is more likely. OR>1 indicates that employment situation "full-time/part-time" is more likely.

^b^Calculated on weighted analysis (weights on age at study, migration background, language region, education, number of children separately for mothers and fathers).

^c^Global p-value calculated from Wald tests.

^d^P-value for interaction between parents of survivors and control parents.

^e^Assumption of proportional odds not met for migration background (mothers and fathers) and language region (mothers only).

^f^OR for those not employed in contrast to those part- or full-time employed. OR<1 indicate that higher values on the explanatory variable increase the likelihood of being not employed. OR>1 indicates that higher values on the explanatory variable increase the likelihood of being part- or full-time employed.

^g^OR for those not employed or part-time employed in contrast to those full-time employed. OR<1 indicate that higher values on the explanatory variable increase the likelihood of being not employed or part-time employed. OR>1 indicates that higher values on the explanatory variable increase the likelihood of being full-time employed.

For fathers, the association with education was no longer statistically significant in multivariable analysis. All other observed associations remained statistically significant (Table 3). Fathers of survivors were more likely to be employed (OR = 1.93, CI:1.02–3.62) compared to control fathers. Among fathers, older age (OR for >50 years = 0.49, CI:0.27–0.88) and having a migration background (OR = 0.23, CI:0.13–0.41) were associated with not being employed. For fathers, associations with socio-demographic variables were similar in both populations (all p for interaction >0.05).

### Associations with socio-demographic and cancer-related variables in parents of survivors

In univariable regression of parents of survivors we found migration background, language region, education, number of children, diagnosis, and relapse status to be associated with either the mothers’ or fathers’ employment situation (Table 4). None of the other cancer-related variables were significantly associated with parents’ employment situation.

**Table 4:** Factors associated with employment in parents of survivors in univariable generalized ordered^a^ logistic regression.

	Parents of survivors (N = 394)					
	Mothers			Fathers		
	OR	95% CI	p-value^b^	OR	95% CI	p-value^b^
**Characteristics of parents**						
*Age at study*			0.639			0.092
<40 years	1.00			1.00		
40–45 years	1.40	0.84–2.35		1.16	0.28–4.83	
45–50 years	1.24	0.72–2.12		1.14	0.26–4.95	
>50 years	1.31	0.46–3.73		0.38	0.10–1.44	
*Migration background*^c^			-			0.264
No	1.00			1.00		
Yes	0.89^d^	0.55–1.43	0.629	0.62	0.27–1.43	
	4.11^e^	2.01–8.42	**<0.001**			
*Language region*^c^			-			0.514
German	1.00			1.00		
French/Italian	1.19^d^	0.73–1.92	0.486	0.76	0.34–1.72	
	2.89^e^	1.43–5.85	**0.003**			
*Education*			**0.012**			**0.042**
Primary	1.00			1.00		
Secondary	1.31	0.80–2.13		3.61	1.02–12.85	
Tertiary	2.92	1.42–5.98		0.61	0.23–1.59	
*Number of children*			**<0.001**			0.317
≤2 children	1.00			1.00		
>2 children	0.46	0.30–0.69		1.53	0.67–3.49	
**Characteristics of the child**						
*Age of child at study*			0.176			0.947
<9 years	1.00			1.00		
9–12 years	1.23	0.67–2.25		0.93	0.29–2.97	
>12 years	1.62	0.94–2.80		1.08	0.37–3.12	
*Age of child at diagnosis*			0.435			0.201
<2 years	1.00			1.00		
2–5 years	0.93	0.59–1.48		1.75	0.64–4.80	
>5 years	1.33	0.78–2.27		0.66	0.27–1.63	
*Diagnosis (ICCC-3)*			**0.024**			0.645
Leukemia	1.00			1.00		
Lymphoma	0.44	0.20–0.96		n.e.^f^		
CNS tumor	1.36	0.74–2.49		1.56	0.41–5.86	
STS/bone tumor	0.78	0.34–1.78		0.57	0.15–2.24	
Other tumors^g^	1.54	0.94–2.54		0.79	0.32–1.93	
*Treatment*			0.226			0.636
Surgery	1.00			1.00		
Chemotherapy	0.66	0.37–1.16		0.98	0.31–3.06	
Radiotherapy	0.94	0.46–1.94		0.54	0.15–2.03	
Stem cell transplantation	0.44	0.16–1.20		0.59	0.10–3.47	
*Time since diagnosis*			0.202			0.354
<8 years	1.00			1.00		
8–11 years	1.24	0.78–1.95		1.89	0.75–4.78	
>11 years	1.64	0.95–2.83		1.60	0.56–4.57	
*Relapse*			0.303			**<0.001**
No	1.00			1.00		
Yes	1.40	0.74–2.67		0.20	0.08–0.47	
*Parent-reported late effects*			0.076			0.163
No	1.00			1.00		
	1.49	0.96–2.33		0.56	0.25–1.26	

CNS, central nervous system; ICCC-3, International Classification of Childhood Cancer -Third Edition; OR, odds ratio; n.e., not estimated; STS, soft tissue sarcoma. bold, p-value lower than 0.05.

^a^Ordering: not employed, part-time, full-time. OR<1 indicates that employment situation "not employed" is more likely. OR>1 indicates that employment situation "full-time/part-time" is more likely.

^b^Global p-value calculated from Wald tests.

^c^Assumption of proportional odds not met for mothers of survivors.

^d^OR for those not employed in contrast to those part- or full-time employed. OR<1 indicate that higher values on the explanatory variable increase the likelihood of being not employed. OR>1 indicates that higher values on the explanatory variable increase the likelihood of being part- or full-time employed.

^e^OR for those not employed or part-time employed in contrast to those full-time employed. OR<1 indicate that higher values on the explanatory variable increase the likelihood of being not employed or part-time employed. OR>1 indicates that higher values on the explanatory variable increase the likelihood of being full-time employed.

^f^Odds ratio not estimated; no variation in outcome.

^g^Other tumors include neuroblastoma, retinoblastoma, renal tumor, hepatic tumor, germ cell tumor, Langerhans cell histiocytosis, other malignant epithelial neoplasms, malignant melanomas, and other or unspecified malignant neoplasms.

In multivariable regression most (except language region) observed associations remained statistically significant (Table 5). We found that mothers with tertiary education (OR = 2.40, CI:1.14–5.07) were more likely to be employed compared to mothers with primary education. Having a migration background increased the likelihood of being full-time employed in mothers of survivors (OR = 3.63, CI:1.71–7.71). Mothers of survivors diagnosed with lymphoma (OR = 0.31, CI:0.13–0.73) and >2 children (OR = 0.48, CI:0.30–0.75) were less likely to be employed (Table 5). Fathers of survivors, who had a relapse (OR = 0.13, CI:0.04–0.36) were less likely to be employed.

**Table 5:** Factors associated with employment in parents of survivors in multivariable generalized ordered^a^ logistic regression.

	Parents of survivors (N = 394)					
	Mothers			Fathers		
	OR	95% CI	p-value^b^	OR	95% CI	p-value^b^
*Migration background*^c^			-			0.093
No	1.00			1.00		
Yes	0.69^d^	0.40–1.17	0.167	0.43	0.16–1.15	
	3.63^e^	1.71–7.71	**0.001**			
*Language region*			0.064			0.682
German	1.00			1.00		
French/Italian	1.58	0.97–2.56		1.22	0.47–3.15	
*Education*			**0.045**			**0.021**
Primary	1.00			1.00		
Secondary	1.45	0.87–2.40		3.28	0.87–12.36	
Tertiary	2.40	1.14–5.07		0.41	0.14–1.16	
*Number of children*			**0.001**			0.583
≤2 children	1.00			1.00		
>2 children	0.48	0.30–0.75		1.30	0.51–3.30	
*Diagnosis (ICCC-3)*			**0.012**			0.470
Leukemia	1.00			1.00		
Lymphoma	0.31	0.13–0.73		n.e.^f^		
CNS tumor	1.30	0.67–2.52		2.23	0.55–8.96	
STS / bone tumor	0.66	0.29–1.54		2.68	0.28–25.87	
Other tumors^g^	1.34	0.79–2.28		0.85	0.32–2.23	
*Relapse*			0.410			**<0.001**
No	1.00			1.00		
Yes	1.35	0.66–2.74		0.13	0.04–0.36	

CNS, central nervous system; ICCC-3, International Classification of Childhood Cancer -Third Edition; OR, odds ratio; n.e., not estimated; STS, soft tissue sarcoma. bold, p-value lower than 0.05.

^a^Ordering: not employed, part-time, full-time. OR<1 indicates that employment situation "not employed" is more likely. OR>1 indicates that employment situation "full-time/part-time" is more likely.

^b^Global p-value calculated from Wald tests.

^c^Assumption of proportional odds not met for mothers of survivors.

^d^OR for those not employed in contrast to those part- or full-time employed. OR<1 indicate that higher values on the explanatory variable increase the likelihood of being not employed. OR>1 indicates that higher values on the explanatory variable increase the likelihood of being part- or full-time employed.

^e^OR for those not employed or part-time employed in contrast to those full-time employed. OR<1 indicate that higher values on the explanatory variable increase the likelihood of being not employed or part-time employed. OR>1 indicates that higher values on the explanatory variable increase the likelihood of being full-time employed.

^f^Not estimated; no variation in outcome.

^g^Other tumors include neuroblastoma, retinoblastoma, renal tumor, hepatic tumor, germ cell tumor, Langerhans cell histiocytosis, other malignant epithelial neoplasms, malignant melanomas, and other or unspecified malignant neoplasms.

## Discussion

This study confirmed the traditional type of parenting roles in Switzerland, and found that this clear distinction between maternal (more often not employed or only part-time employed) and paternal roles (more often full-time employed) became even more pronounced in parents of survivors. However, no differences between mothers of survivors and mothers in the general population were found after adjusting for socio-demographic variables in multivariable analyses. Associations with socio-demographic variables were similar in both populations. Having >2 children had a stronger effect on the employment situation of mothers of survivors. Among parents of survivors, education, number of children, diagnosis of lymphoma, and relapse status were the most important determinants of employment.

Previous studies suggested that disruptions of parents’ employment situation are most frequent shortly after diagnosis and during treatment [1, 3, 5, 7–11]. However, we found that employment situation differed between parents of survivors and the general population even long after treatment ended. These findings are different to results from a Norwegian study, which showed that parents’ long-term employment situation was not affected by childhood cancer [4]. Another study in Canada showed that childhood cancer only temporarily affected parental employment and most families were able to return to work within 5 years after diagnosis [1]. These different findings may be partially explained by country-specific differences in labor market policies and social security systems. The relatively high proportion of mothers of survivors not being employed in our study may be explained by lack of opportunities for paid leave to care for seriously ill children, or systems facilitating return to work after the child’s treatment. Parents caring for ill children have the right for 2–3 days off work per year in Switzerland. However, the average time parents need for caretaking of children with cancer has been estimated to add up to approximately 240 working days in Switzerland [27]. Therefore, more flexible working conditions during the child’s treatment combined with more comprehensive support for families during and after a diagnosis of childhood cancer may be advocated, especially for mothers of survivors. The need for family friendly policies to provide a supportive environment for families having a child with cancer was also emphasized by a qualitative study in Australia [12]. They concluded that parents of survivors experience substantial work-family conflicts also after the child’s recovery [12].

Traditionally in Switzerland, mothers are the primary caregiver and remaining employed may be more difficult while caring for a child with cancer [4, 8]. However, in multivariable analysis the population variable was no longer significant indicating that other factors such as education or the number of children are more important for determining the maternal employment situation rather than being a mother of a survivor. Conversely, we showed that fathers of survivors were more often employed full-time. It has been suggested, that fathers of survivors may work more to compensate for reductions of working hours and income of mothers [4]. However, more information is needed to understand why families of children with cancer show such employment patterns (e.g. what are the needs at home, factors within the work place, etc.).

The observed gender differences in our study reflect the traditional view of gendered parenting roles in Switzerland, which we found to be even more pronounced in parents of survivors. However, we can only hypothesize about reasons for this pattern. A previous study indicated that mothers may decide not to be employed due to other priorities, e.g. spending more time with the family after the cancer experience [5]. Another study in Japan showed that 70% of mothers reported that they lost work motivation after diagnosis [11]. Other reasons may be lost or declined job opportunities and promotions [1, 10]. However, limited data availability did not allow to investigate the parents’ employment situation prior to the child’s cancer diagnosis or along the disease trajectory.

In our study, mothers with >2 children were less likely to be employed which may be explained by higher caregiving demands. Having more siblings exposed to the experience of childhood cancer may increase the need for attention from parents as siblings of children with cancer may experience post-traumatic stress symptoms, negative emotional reactions or poor quality of life [28]. The absence of such an association among fathers may be a result of the mothers’ role as the primary caregiver. The stronger association among mothers of survivors compared to mothers of the general population may intuitively be explained by higher caregiving demands going along with the cancer diagnosis.

We also found that mothers with higher education were more likely to be employed. In a study in Japan, maternal employment was not affected by education [11]. However, a study in Norway found that mothers with lower education were less likely to leave employment when having a child with cancer [4]. The authors assumed, that jobs requiring lower education are less flexible in terms of working hours [4], which may also partially explain our findings. However, people with lower education may also have less financial resources to cope with the complex and enduring problems of having a child with cancer, whereas those with higher education might be more likely to afford external childcare for example.

Having a migration background was differently associated with the employment situation of mothers and fathers. For both, mothers and fathers, we observed that having a migration background increased the likelihood of not being employed. This is in line with national estimates generally showing higher unemployment rates among foreigners in Switzerland [29]. However, for mothers with a migration background we also observed that they were more likely to be full-time employed rather than only part-time. This is similar to findings among women in the general population of Switzerland [29].

In terms of cancer-related variables, we found that mothers of survivors diagnosed with lymphoma (and less pronounced also mothers of survivors diagnosed with soft tissue sarcoma/bone tumor) and fathers of survivors, who had had a cancer relapse, were less likely to be employed. The relatively intensive treatment following the diagnosis of lymphoma may partially explain reduced long-term employment among mothers. In our study, all fathers of survivors diagnosed with lymphoma were full-time employed, possibly to compensate reduced employment of mothers. Cancer relapse may impact the work ability of fathers due to higher caregiving demands.

A decrease in parents’ working hours was also associated with more intensive treatment in another study [10]. A study in Sweden found that parents with children younger than 10 years and diagnosed with leukemia were more likely to leave jobs [1]. However, two studies conducted in Japan and Norway showed that diagnosis and age at diagnosis were not associated with parents’ likeliness to be employed [4, 11]. This may be partially explained by differences related to country-specific labor market policies or social security systems.

### Limitations and strengths

A limitation is the relatively small sample size of parents of survivors, which reduced the accuracy of effect estimates in the analyses. Our sample was restricted to parents of survivors aged 5–15 years. Caregiving demands of parents of younger survivors may be higher, which may overestimate the general impact on parent’s employment. The questionnaire focused on the health state of the survivor, therefore no in-depth questions on parents’ employment preferences (e.g. if they were actively looking for work, or whether parents were satisfied with their current employment situation), support from any form of governmental assistance, or time needed for caregiving were available. The study design did not allow us to investigate parents’ employment situation longitudinally along the child’s illness trajectory and consequently we do not know whether parents employment status changed as a result of the cancer diagnosis, treatment and cure. Contrary to parents of survivors, the sample of controls consisted of independent samples of mothers and fathers. Therefore, selection bias due to participation may be different in control parents compared to survivors’ parents. However, we minimized this type of bias by weighting the control population separately for mothers and fathers. We had no information about potential chronic health conditions in children of control parents. Therefore, differences between parents of survivors and parents of healthy children might be underestimated. Concerns also arise regarding the response rate of 53% in the Swiss Health Survey 2012. The analysis of a subsample of the Swiss Health Survey in 2007 showed that participants were more likely to have a higher socio-economic status, better subjective health and were more likely to be Swiss nationals than non-participants [30]. However, it remains unclear if this is also true for the Swiss Health Survey from 2012.

A major strength of the present study is the population-based sampling approach and the high response rate among parents of survivors (75%) limiting the presence of selection bias. Additionally, clinical information from medical records was derived from the Swiss Childhood Cancer Registry. The use of similar inclusion criteria and the same questions on employment and socio-demographic characteristics for parents of survivors and the general population enabled an adequate comparison. Comparability was further maximized by weighting the sample of the general population according to parents of survivors separately for mothers and fathers.

However, employment patterns and how having a child with a chronic disease affects parents’ employment situation strongly depends on country-specific circumstances. Therefore, generalizability of our findings is limited and our findings may only apply to countries with similar employment rates, labor market policies as well as social welfare and health care systems.

## Conclusions

The long-term employment situation of parents of survivors reflects more traditional roles of parenting with mothers being more often not employed and fathers being more often full-time employed. More flexible working conditions during the child’s treatment combined with facilitating return to work may be advocated to support long-term employment opportunities especially among mothers of survivors. Specific support for parents with low education, additional children, and whose child had a more severe cancer disease could improve their long-term employment situation.

## Supporting Information

S1 Fig Participants of the Swiss Health Survey 2012. Flow diagram of the Swiss Health Survey 2012 starting from those randomly selected for the survey to those included in the analysis.
(PDF)

S2 Fig Employment situation of parents of survivors stratified by diagnostic group compared to control parents. Employment situation of parents of survivors (CCS) stratified by diagnostic group compared to control parents (SHS). The numbers in the figure represent the proportion of mothers and fathers who reported the respective employment situation. *Weighted proportions and numbers for control mothers and control fathers according to age at study, migration background, language region, education, and number of children of mothers and fathers of survivors. †Other tumors include neuroblastoma, retinoblastoma, renal tumor, hepatic tumor, germ cell tumor, Langerhans cell histiocytosis, other malignant epithelial neoplasms, malignant melanomas, and other or unspecified malignant neoplasms. Abbreviations: CCS, childhood cancer survivors; CNS, central nervous system; SHS, Swiss Health Survey; STS, soft tissue sarcoma.
(PDF)

S1 Table Socio-demographic characteristics of parents of survivors and control parents. SD, standard deviation. bold, p-value lower than 0.05. ^a^Restricted to couples having children aged 5–15 years. ^b^Percentages are based upon available data for each variable. ^c^P-value calculated from chi-square statistics or ttest comparing mothers of survivors to control mothers. ^d^P-value calculated from chi-square statistics or ttest comparing fathers of survivors to control fathers.
(PDF)
