# Supplementary material for: Employment Situation of Parents of Long-Term Childhood Cancer Survivors
Source: PLoS One. 2016 Mar 18;11(3):e0151966. doi: 10.1371/journal.pone.0151966 (PMC4798766; doi:10.1371/journal.pone.0151966)
Supplement: S1 Fig — Flow diagram of the Swiss Health Survey 2012 starting from those randomly selected for the survey to those included in the analysis. (PDF) [file pone.0151966.s001.pdf]

S1 Fig. Participants of the Swiss Health Survey 2012

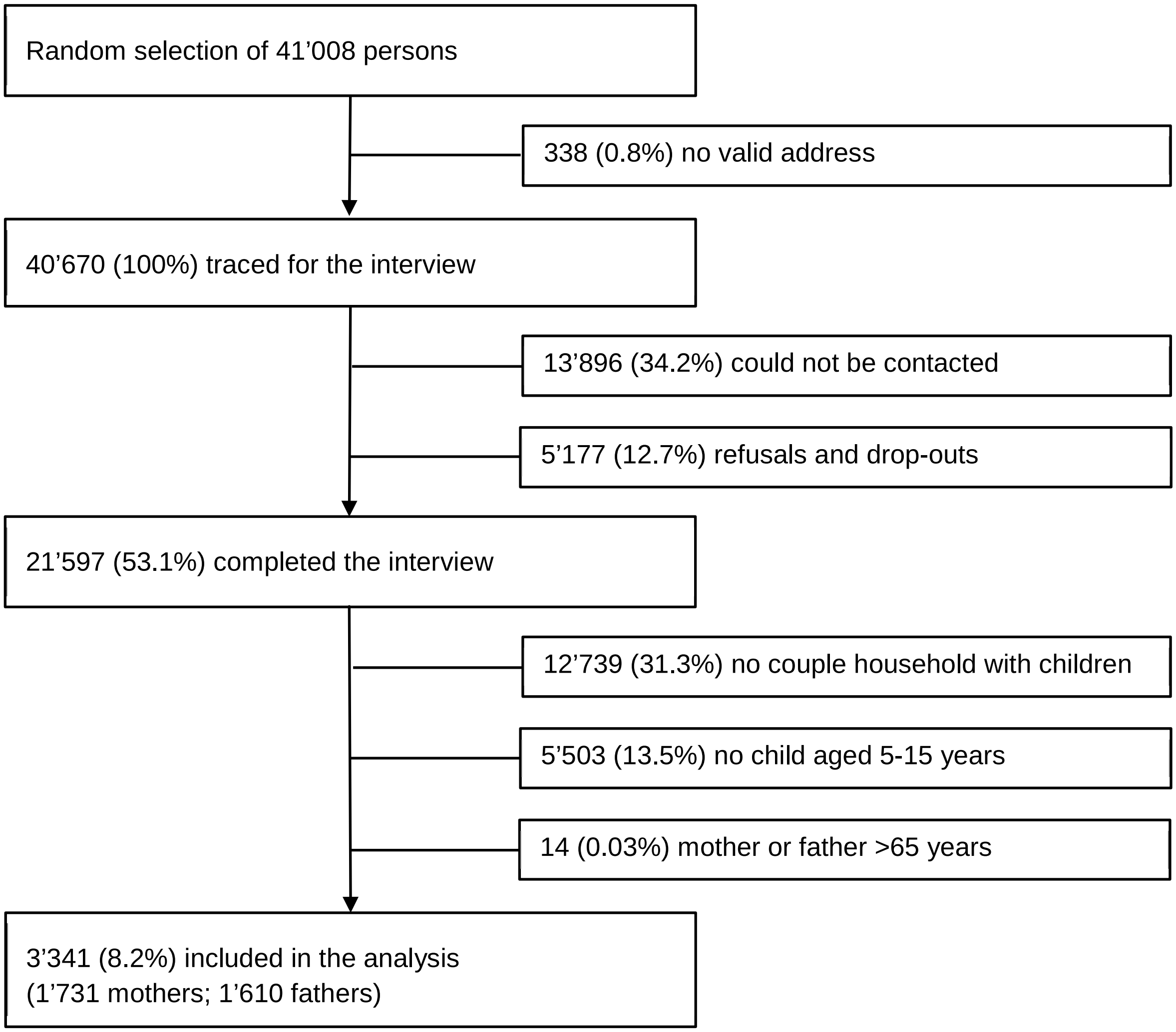

Flow diagram of the Swiss Health Survey 2012 starting from those randomly selected for the survey to those included in the analysis.
