# Supplementary material for: Employment Situation of Parents of Long-Term Childhood Cancer Survivors
Source: PLoS One. 2016 Mar 18;11(3):e0151966. doi: 10.1371/journal.pone.0151966 (PMC4798766; doi:10.1371/journal.pone.0151966)
Supplement: S2 Fig — Employment situation of parents of survivors (CCS) stratified by diagnostic group compared to control parents (SHS). The numbers in the figure represent the proportion of mothers and fathers who reported the respective employment situation. *Weighted proportions and numbers for control mothers and control fathers according to age at study, migration background, language region, education, and number of children of mothers and fathers of survivors. †Other tumors include neuroblastoma, retinoblastoma, renal tumor, hepatic tumor, germ cell tumor, Langerhans cell histiocytosis, other malignant epithelial neoplasms, malignant melanomas, and other or unspecified malignant neoplasms. Abbreviations: CCS, childhood cancer survivors; CNS, central nervous system; SHS, Swiss Health Survey; STS, soft tissue sarcoma. (PDF) [file pone.0151966.s002.pdf]

S2 Fig. Employment situation of parents of survivors stratified by diagnostic group compared to control parents

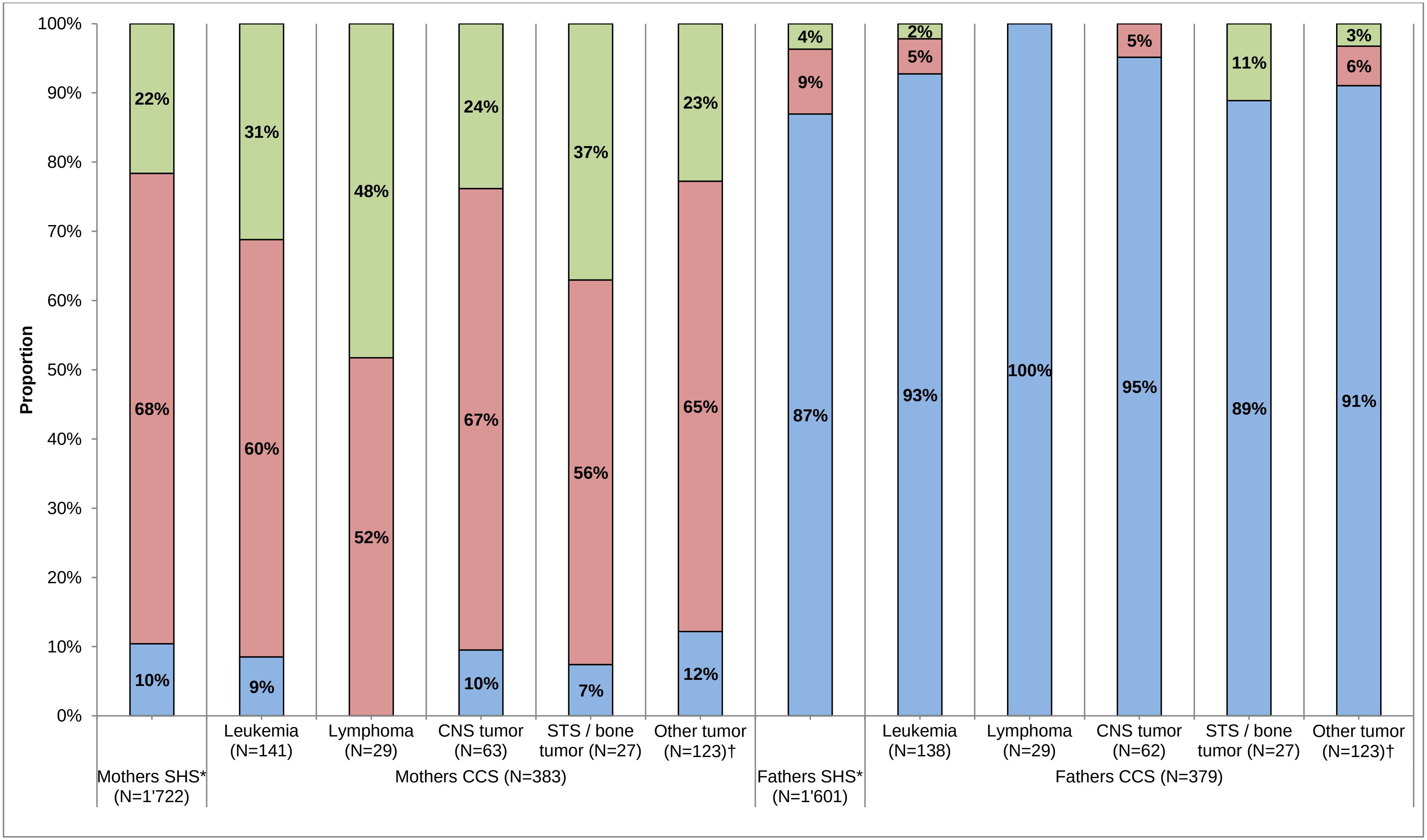

Employment situation of parents of survivors (CCS) stratified by diagnostic group compared to control parents (SHS). The numbers in the figure represent the proportion of mothers and fathers who reported the respective employment situation.

*Weighted proportions and numbers for control mothers and control fathers according to age at study, migration background, language region, education, and number of children of mothers and fathers of survivors.

†Other tumor include neuroblastoma, retinoblastoma, renal tumor, hepatic tumor, germ cell tumor, Langerhans cell histiocytosis, other malignant epithelial neoplasms, malignant melanomas, and other or unspecified malignant neoplasms.

Abbreviations: CCS, childhood cancer survivors; CNS, central nervous system; SHS, Swiss Health Survey; STS, soft tissue sarcoma.

Legend:

- Not employed
- Part-time
- Full-time
