# Supplementary material for: Employment Situation of Parents of Long-Term Childhood Cancer Survivors
Source: PLoS One. 2016 Mar 18;11(3):e0151966. doi: 10.1371/journal.pone.0151966 (PMC4798766; doi:10.1371/journal.pone.0151966)
Supplement: S1 Table — SD, standard deviation. bold, p-value lower than 0.05. aRestricted to couples having children aged 5–15 years. bPercentages are based upon available data for each variable. cP-value calculated from chi-square statistics or ttest comparing mothers of survivors to control mothers. dP-value calculated from chi-square statistics or ttest comparing fathers of survivors to control fathers. (PDF) [file pone.0151966.s003.pdf]

S1 Table. Socio-demographic characteristics of parents of survivors and control parents

	Parents of survivors (N=394)				Control parents[a] (N=3’341)					
	Mothers		Fathers		Mothers (N=1’731)		Fathers (N=1’610)			
	n	%[b]	n	%[b]	n	%[b]	n	%[b]	p mothers[c]	p fathers[d]
Age at study									0.080	**<0.001**
<40 years	110	29.0	55	14.6	619	35.8	370	23.0		
40-45 years	140	36.9	130	34.4	572	33.0	460	28.6		
45-50 years	114	30.1	109	28.8	465	26.9	544	33.8		
>50 years	15	4.0	84	22.2	75	4.3	236	14.7		
Migration background									**0.013**	**<0.001**
No	276	70.1	299	75.9	1’097	63.5	1’073	66.7		
Yes	118	30.0	95	24.1	632	36.6	536	33.3		
Language region									**0.048**	**0.037**
German	271	69.0	271	69.0	1’102	63.7	1’020	63.4		
French/Italian	122	31.0	122	31.0	629	36.3	590	36.7		
Education									0.252	0.258
Primary	242	64.2	178	48.6	1’112	64.4	733	45.8		
Secondary	95	25.2	132	36.1	387	22.4	566	35.3		
Tertiary	40	10.6	56	15.3	229	13.3	303	18.9		
Number of children									**<0.001**	**0.001**
≤2 children	226	57.4	226	57.4	1’159	67.0	1’070	66.5		
>2 children	168	42.6	168	42.6	572	33.0	540	33.5		
	Mean	SD	Mean	SD	Mean	SD	Mean	SD		
Age at study	42.7	4.8	45.8	5.8	41.4	5.8	44.3	6.3	**<0.001**	**<0.001**

SD, standard deviation. bold, p-value lower than 0.05.

[a]Restricted to couples having children aged 5-15 years.
[b]Percentages are based upon available data for each variable.
[c]P-value calculated from chi-square statistics or ttest comparing mothers of survivors to control mothers.
[d]P-value calculated from chi-square statistics or ttest comparing fathers of survivors to control fathers.
